# Regional differences in the utilization and outcomes of cerebral embolic protection during transcatheter aortic valve replacement: an analysis of the National Inpatient Sample from 2017 through 2019

**DOI:** 10.57264/cer-2023-0010

**Published:** 2023-09-19

**Authors:** Elisa M Amoroso

**Affiliations:** 1 Department of Health Services Administration, Xavier University, Cincinnati, OH 45207, USA

**Keywords:** outcomes, sentinel, TAVR, utilization

## Abstract

**Aim:**

To evaluate the utilization and outcomes of cerebral embolic protection (CEP) during transcatheter aortic valve replacement (TAVR) by USA region, using discharge data from the National Inpatient Sample (NIS), Healthcare Cost and Utilization Project (HCUP), Agency for Healthcare Research and Quality.

**Patients & methods:**

All TAVR discharge encounters from June 2017–2019 were included in the analysis. Discharge encounters with bicuspid anatomy were excluded. Regional CEP utilization rates were reported. For TAVR cases performed with the Sentinel CEP device (Boston Scientific, MA, USA), multivariable logistic regression was performed to model regional differences in TAVR outcomes including: stroke, transient ischemic attack (TIA), stroke/TIA combined, and in-hospital all-cause mortality. Generalized linear regression models were used to assess regional differences in length of stay (LOS) and hospital charges.

**Results:**

The Northeast had the greatest overall CEP utilization rate (11.3%), followed by the Midwest (11.1%), West (8.7%), then South (3.1%). Compared with the Northeast, the South was associated with a lower risk of stroke (OR: 0.267, 95% CI: 0.106–0.673; p = 0.005), and the West a higher risk of stroke (OR: 1.583, 95% CI: 1.044–2.401; p = 0.031). Compared with the Northeast, the West was associated with a higher risk of stroke/TIA combined (OR: 1.618, 95% CI: 1.107–2.364; p = 0.013). Compared with the Northeast, the Midwest (OR: 4.501, 95% CI: 2.229–9.089; p < 0.001) and West (OR: 5.316, 95% CI: 2.611–10.824; p < 0.001) were associated with a higher risk of in-hospital all-cause mortality. Adjusted charges and LOS were highest in the West.

**Conclusion:**

Within the USA, there are regional differences in the utilization and outcomes of CEP use during TAVR. To prevent regional disparities and ensure consistent quality of care in the USA, further research is needed to determine what variable(s) may be responsible for regional differences in TAVR outcomes, with or without CEP.

## Plain language summary

**Regional differences in the use & outcomes of cerebral embolic protection during transcatheter aortic valve replacement:**

**What is this article about?:**

The use of cerebral embolic protection (CEP), to mitigate the risk of stroke, during transcatheter aortic valve replacement (TAVR) remains low despite commercial availability since 2017. This study assessed USA regional differences in the use and outcomes of CEP during TAVR.

**What were the results?:**

The Northeast had the greatest overall CEP utilization rate (11.3%), followed by the Midwest (11.1%), West (8.7%), then South (3.1%). Compared with the Northeast, the South was associated with a lower risk of stroke, and the West a higher risk of stroke. Compared with the Northeast, the West was associated with a higher risk of stroke/TIA combined. Compared with the Northeast, the Midwest and West were associated with a higher risk of in-hospital all-cause mortality.

**What do the results mean?:**

To prevent regional disparities and ensure consistent quality of care in the USA, further research is needed to determine what variable(s) may be responsible for regional differences in TAVR outcomes, with or without CEP.

## Tweetable abstract

Within the USA, there are regional differences in the utilization and outcomes of cerebral embolic protection use during transcatheter aortic valve replacement. #TAVR #Sentinel

It is estimated that by 2025, transcatheter aortic valve replacement (TAVR) will account for 75% of all aortic valve replacements [1,2]. Despite the overall success of TAVR and improvements in device technology and operator technique, approximately 2 in 100 patients will suffer a periprocedural stroke [3,4]. The cerebral embolic protection (CEP) device was developed to capture or deflect emboli from entering cerebral circulation during the TAVR procedure [5,6]. The Sentinel CEP device (Boston Scientific, MA, USA) received approval for use from the US FDA in June 2017 and, to date, remains the only FDA approved CEP device for use during TAVR.

In TAVR cohorts, for patients that experience a periprocedural stroke, the 30-day mortality rate is significantly higher than the rate for those who do not experience stroke [7,8]. TAVR patients that suffer from stroke are also at high risk for severe morbidity [5,8–10]. Ischemic stroke experienced during the index hospitalization is associated with a 32% increase in costs [11]. Despite the clinical and economic burden of disease, CEP is utilized, to varying degrees, by just over 25% of TAVR centers [3,12]. Data from the Transcatheter Valve Therapy (TVT) Registry from January 2018 through December 2019 revealed that the Sentinel CEP device (Boston Scientific) was utilized in 13% of all cases by the end of the fourth quarter of 2019 [3]. It has been speculated that the cost of the device, estimated at $2400, has been a barrier to adoption, as many TAVR centers already operate on minimal profit margins or lose money [4,13–15]. However, the Centers for Medicare & Medicaid Services (CMS) approved a new technology add-on payment for the CEP device, in the amount of $1400, beginning 01 October 2018 [11,15,16]. Additionally, the American Medical Association established a CPT code for physician use of the device. The payment rate is $131 and varies based on region [17].

While ongoing clinical studies are assessing the efficacy of the Sentinel and other investigational CEP devices, analysis on utilization trends remains sparse, despite commercial availability since 2017 and reimbursement since 2018. Further, regional differences are often unexplored in outcomes research, which is a missed opportunity to explore potential disparities in clinical care. The objective of this analysis was to evaluate the utilization and outcomes of CEP during TAVR by USA region, using discharge data from the National Inpatient Sample (NIS), Healthcare Cost and Utilization Project (HCUP), Agency for Healthcare Research and Quality.

## Material & methods

### Data

Data was obtained from the National Inpatient Sample (NIS), Healthcare Cost and Utilization Project (HCUP), Agency for Healthcare Research and Quality. The NIS database captures a 20% stratified sample of discharges from USA community hospitals, excluding rehabilitation and long-term acute care facilities. The NIS is the largest public database in the USA to produce regional and national estimates of utilization, access, charges and quality among all USA payer groups [18].

NIS data from June 2017 through 2019 was used for this analysis. This time period captures the commercial availability of the Sentinel CEP device (Boston Scientific), June 2017, through the current date of available NIS data, 2019. Each discharge encounter contains up to 40 diagnoses and 25 procedures. The patient population, covariates, and outcomes of interest were identified using the International Classification of Diseases 10th Revision (ICD-10) Clinical Modification of medical diagnoses and Procedure Coding System (ICD-10-CM/PCS) for inpatient procedures [19]. Discharge weights were applied to the sample as required per HCUP regulations to produce regional estimates. Discharge weighting is necessary due to the NIS data being a stratified sample [20]. HCUP data is publicly available and HIPAA compliant [21]. This study received a waiver of IRB oversight from the Xavier University IRB.

### Methods

The analysis was conducted from the perspective of the hospital system. Discharge encounters with a primary ICD-10-PCS of 02RF38Z, percutaneous TAVR, or 02RF38H, transapical TAVR, were included. Bicuspid anatomy was excluded from the analysis. These discharge encounters were identified via ICD-10-CM of Q230, congenital stenosis of the aortic valve, or Q231, congenital insufficiency of the aortic valve [19]. Bicuspid valves are often associated with having heavier calcification than tricuspid valves. They have also been associated with unfavorable TAVR outcomes, including lower rates of device success and higher rates of paravalvular leak and 30-day stroke [5,22–24]. TAVR cases performed with the Sentinel CEP device (Boston Scientific) were identified using ICD-10-PCS of X2A5312 [17]. USA regions were defined as: Northeast, Midwest, South, and West [20]. The outcomes explored in this study were: CEP utilization rates, stroke (including all ICD-10-CM codes for nontraumatic subarachnoid hemorrhage, nontraumatic intracerebral hemorrhage, and cerebral infarction), transient ischemic attack (TIA), stroke/TIA combined, in-hospital all-cause mortality, length of stay (LOS), and charges.

### Statistical analysis

Categorical variables were expressed as counts and percentages. Outcome variables were analyzed for missing data. Binary outcome variables (i.e., utilization, stroke, TIA, and in-hospital all-cause mortality) were expressed as counts and rates. For nominal variables, differences between groups were compared using the Pearson chi-square test. Metric variables (i.e., age, LOS, charges, etc.) were expressed as mean and standard deviation and median and interquartile range (IQR). For metric variables, differences between groups were compared using One-Way ANOVA and interpreted via the Welch test due to a violation of the homogeneity of variance assumption.

Multivariable logistic regression was performed to model regional differences in TAVR outcomes including: stroke, TIA, stroke/TIA combined, and in-hospital all-cause mortality. The results of the multivariable logistic regression models were reported as odds ratios, 95% confidence intervals for the odds ratios, and p-values. Generalized linear regression models were used to assess regional differences in LOS and charges. The generalized linear models were run with log links and gamma distributions due to the positive skew of the outcome data. Model estimates with 95% confidence intervals were reported. Each model was adjusted for independent covariates selected *a priori* from a literature review, including the research performed by Elixhauser *et al.* to identify comorbidities of significance and in consideration of the Society of Thoracic Surgeons (STS) risk factors for operative mortality and morbidity [25–27]. Sensitivity analyses were performed. For the first sensitivity analysis, discharge encounters associated with a history of prosthetic heart valves were excluded from the dataset. The objective of this sensitivity analysis was to assess for differences in outcomes by excluding the valve-in-valve cases. A second sensitivity analysis was performed utilizing the TAVR cohort, to determine if use of the Sentinel CEP device (Boston Scientific) was a significant predictor of stroke/TIA combined, in-hospital all-cause mortality, and LOS. An α level of 0.05 was used to determine statistical significance. Statistical analysis was performed using IBM SPSS Statistics version 27.0.

## Results

**Figure 1.**
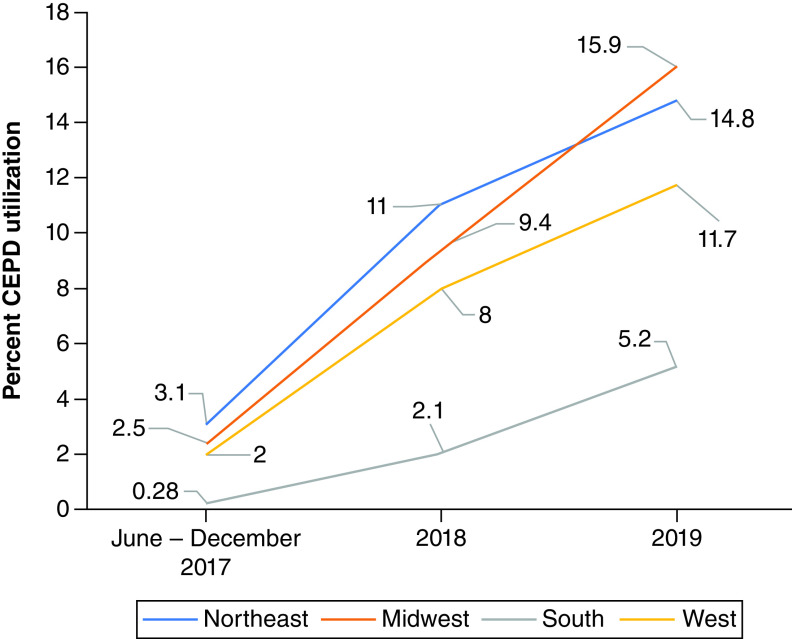
Percent CEP utilization by region and year.

Baseline characteristics and summary statistics were reported by USA region (Table 1). From June 2017–2019, there were 145,800 weighted discharge encounters in the TAVR cohort and 11,630 (8%) weighted discharge encounters in the cohort of TAVR cases performed with the Sentinel CEP device. The majority of TAVR cases were performed in the South (33.5%). The Northeast (23.3%), Midwest (23.1%), and West (20.1%) had similar proportions of TAVR cases performed. The Northeast had the greatest overall CEP utilization rate (11.3%); the rate in the Midwest (11.1%) was similar, while rates in the West (8.7%) and South (3.1%) were lower. By 2019, the Midwest had the highest rate of CEP utilization (15.9%), followed by the Northeast (14.8%), West (11.7%), then South (5.2%) (Figure 1). Overall and yearly regional differences in CEP utilization were statistically significant. Each region experienced a year-over-year increase in CEP utilization. The largest year-over-year and study period percent increase in CEP utilization was observed in the South. For TAVR cases performed with the Sentinel CEP device, there were statistically significant differences between regions in demographics, comorbidities, medical history, and outcomes (Table 1). After analyzing outcome variables for missing data, missing values were identified for charges (n = 45). These discharge encounters were not excluded from the dataset as they accounted for 0.39% of the data. Missing values were not identified for other outcomes of interest. The baseline rate of stroke/TIA combined and in-hospital all-cause mortality were highest in the West, at 2.2% and 1.4%, respectively (Table 1). The baseline rate of stroke/TIA combined was 1.6% in the Northeast, 1.3% in the Midwest, and 1% in the South. The Midwest region had a baseline rate of in-hospital all-cause mortality of 1.1%, slightly lower than the West. The baseline rate of in-hospital all-cause mortality was lower in the Northeast and South; similar rates were observed in these two regions (Table 1).

**Table 1.** Summary statistics by region.

	Northeast	Midwest	South	West	p-value
Utilization					
Overall TAVR cases	34,000 (23.3%)	33,745 (23.1%)	48,815 (33.5%)	29,240 (20.1%)	p < 0.001
Overall TAVR cases with CEP use	3830 (11.3%)	3735 (11.1%)	1530 (3.1%)	2535 (8.7%)	p = 0.000

TAVR cases by year (n)/CEPD utilization (%)					
June – December 2017	6595 (3.1%)	6445 (2.5%)	9055 (.28%)	5155 (2%)	p < 0.001
2018	11,580 (11%)	11,810 (9.4%)	17,765 (2.1%)	10,390 (8%)	p < 0.001
2019	15,825 (14.8%)	15,490 (15.9%)	21,995 (5.2%)	13,695 (11.7%)	p < 0.001

Demographics					
Age, Mean (SD)	80 (8)	79 (8)	78 (8)	78 (10)	p < 0.001^†^
Age, Median (IQR)	81 (75–87)	80 (74–85)	78 (72–84)	79 (72–85)	
Female	1640 (42.8%)	1600 (42.8%)	685 (44.8%)	1020 (40.2%)	p = 0.031
Male	2190 (57.2%)	2135 (57.2%)	2845 (55.2%)	1515 (59.8%)	

Comorbidities					
Carotid artery disease	170 (4.4%)	145 (3.9%)	80 (5.2%)	120 (4.7%)	p = 0.138
Congestive heart failure	2930 (76.5%)	2745 (73.5%)	1240 (81.0%)	1990 (78.5%)	p < 0.001
Peripheral vascular disease	265 (6.9%)	225 (6%)	135 (8.8%)	160 (6.3%)	p = 0.002
Diabetes	1350 (35.2%)	1395 (37.3%)	585 (38.2%)	915 (36.1)	p = 0.116
Chronic kidney disease	1110 (29%)	1330 (35.6%)	530 (34.6%)	830 (32.7%)	p < 0.001
Obesity	625 (16.3%)	825 (22.1%)	285 (18.6%)	565 (22.3%)	p < 0.001
Hypertension	670 (17.5%)	710 (19.0%)	215 (14.1%)	340 (13.4%)	p < 0.001
Atrial fibrillation or flutter	1440 (37.6%)	1515 (40.6%)	545 (35.6%)	1005 (39.6%)	p = 0.002
Chronic pulmonary disease	570 (14.9%)	555 (14.9%)	285 (18.6%)	425 (16.8%)	p = 0.001

Coagulopathy	^‡^	^‡^	^‡^	^‡^	p = 0.001
Anemia	690 (18.0%)	380 (10.2%)	180 (11.8%)	285 (11.2%)	p < 0.001

Medical History					
History of prior MI	415 (10.8%)	595 (15.9%)	205 (13.4%)	250 (9.9%)	p < 0.001
History of PCI	700 (18.3%)	855 (22.9%)	310 (20.3%)	365 (14.4%)	p < 0.001
History of CABG	450 (11.7%)	650 (17.4%)	225 (14.7%)	270 (10.7%)	p < 0.001
History of stroke or TIA	485 (12.7%)	500 (13.4%)	205 (13.4%)	340 (13.4%)	p = 0.750
History of smoking	1510 (39.4%)	1375 (36.8%)	525 (34.3%)	910 (35.9%)	p = 0.001
History of prosthetic heart valve	50 (1.3%)	80 (2.1%)	20 (1.3%)	25 (1%)	p = 0.001

Outcomes					
Stroke	^‡^	^‡^	^‡^	^‡^	p = 0.001
TIA	^‡^	^‡^	^‡^	^‡^	p < 0.001
Stroke/TIA, combined	60 (1.6%)	50 (1.3%)	15 (1.0%)	55 (2.2%)	p = 0.013
In-hospital death	^‡^	40 (1.1%)	^‡^	35 (1.4%)	p < 0.001
Length of stay, Median (IQR)	2 (1–4) days	2 (1–3) days	2 (1–4) days	2 (1–3) days	p < 0.001^†^
Charges, Median (IQR)	$178,723 ($126,586–288,923)	$158,829 ($127,550–200,025)	$181,587 ($133,211–245,774)	$205,532 ($144,783–310,521)	p = 0.000^†^

† One-way ANOVA, otherwise Pearson chi-square.

‡ Counts are blinded to maintain confidentiality.

Multivariable logistic regression revealed that compared with the Northeast region, TAVR cases performed with CEP in the South were associated with a lower risk of stroke, and in the West, a higher risk of stroke. Compared with the Northeast region, patients in the South were 73% less likely to experience a stroke (OR: 0.267, 95% CI: 0.106–0.673; p = 0.005), and patients in the West were 58% more likely to experience a stroke (OR: 1.583, 95% CI: 1.044–2.401; p = 0.031). None of the USA regions were statistically significant predictors for TIA. For TAVR cases performed with CEP, the West region had a higher risk of stroke/TIA combined (Table 2). Compared with the Northeast region, patients in the West were 62% more likely to experience a stroke or TIA (OR: 1.618, 95% CI: 1.107–2.364; p = 0.013) (Table 2). For TAVR cases performed with CEP, the Midwest and West regions were associated with a higher risk of in-hospital all-cause mortality (Table 3). Compared with the Northeast region, patients in the Midwest were 350% more likely to experience in-hospital death (OR: 4.501, 95% CI: 2.229–9.089; p < 0.001), and patients in the West were 432% more likely to experience in-hospital death (OR: 5.316, 95% CI: 2.611–10.824; p < 0.001).

**Table 2.** Unadjusted and adjusted odds ratio for stroke/TIA combined.

Predictor	Unadjusted			Adjusted		
	OR	95% CI	p-value	OR	95% CI	p-value
Region, Midwest	0.853	0.584–1.244	0.408	0.931	0.632–1.372	0.717
Region, South	0.622	0.352–1.099	0.102	0.697	0.381–1.208	0.188
Region, West	1.393	0.963–2.016	0.078	1.618	1.107–2.364	0.013

Reference: Region, Northeast.

The adjusted model was adjusted for: age, gender, carotid artery disease, congestive heart failure, peripheral vascular disease, diabetes, chronic kidney disease, obesity, hypertension, atrial fibrillation or flutter, chronic pulmonary disease, coagulopathy, anemia, history of prior myocardial infarction, history of percutaneous coronary intervention, history of coronary bypass graft surgery, history of stroke or TIA, history of smoking.

**Table 3.** Unadjusted and adjusted odds ratio for in-hospital all-cause mortality.

Predictor	Unadjusted			Adjusted		
	OR	95% CI	p-value	OR	95% CI	p-value
Region, Midwest	4.135	2.065–8.281	<0.001	4.501	2.229–9.089	<0.001
Region, South	1.252	0.427–3.670	0.682	1.216	0.412–3.589	0.723
Region, West	5.348	2.644–10.819	<0.001	5.316	2.611–10.824	<0.001

Reference: Region, Northeast.

The adjusted model was adjusted for: age, gender, carotid artery disease, congestive heart failure, peripheral vascular disease, diabetes, chronic kidney disease, obesity, hypertension, atrial fibrillation or flutter, chronic pulmonary disease, coagulopathy, anemia, history of prior myocardial infarction, history of percutaneous coronary intervention, history of coronary bypass graft surgery, history of stroke or TIA, history of smoking.

Generalized linear regression models revealed that all USA regions were significant predictors for charges, and all regions except for the South were significant predictors for LOS. After model adjustments were made, the mean adjusted charge (Table 4) was highest in the West (USD409,361, 95%CI USD373,254–USD448,961), followed by the Northeast (USD289,523, 95% CI USD264,150-USD317,333), South (USD263,668, 95% CI USD240,272–USD289,342), then Midwest (USD212,181, 95% CI USD193,611–USD232,533). The mean adjusted LOS (Table 5) was the longest in the West (5 days, 95% CI 4–6 days). All other regions had a mean adjusted LOS of 4 days (95% CI 4–5 days).

**Table 4.** Adjusted charge estimates.

Factor	Adjusted mean charges	95% Confidence Interval
Region, Northeast	USD289,523	USD264,150-$317,333
Region, Midwest	USD212,181	USD193,611-$232,533
Region, South	USD263,668	USD240,272-$289,342
Region, West	USD409,361	USD373,254-$448,961

The model was adjusted for: age, sex, carotid artery disease, congestive heart failure, peripheral vascular disease, diabetes, chronic kidney disease, obesity, hypertension, atrial fibrillation or flutter, chronic pulmonary disease, coagulopathy, anemia, history of prior myocardial infarction, history of percutaneous coronary intervention, history of coronary bypass graft surgery, history of stroke or TIA, history of smoking.

**Table 5.** Adjusted LOS estimates.

Factor	Adjusted mean LOS	95% Confidence Interval
Region, Northeast	4 days	4–5 days
Region, Midwest	4 days	4–5 days
Region, South	4 days	4–5 days
Region, West	5 days	4–6 days

The model was adjusted for: age, sex, carotid artery disease, congestive heart failure, peripheral vascular disease, diabetes, chronic kidney disease, obesity, hypertension, atrial fibrillation or flutter, chronic pulmonary disease, coagulopathy, anemia, history of prior myocardial infarction, history of percutaneous coronary intervention, history of coronary bypass graft surgery, history of stroke or TIA, history of smoking.

LOS: Length of stay.

After excluding discharge encounters associated with a history of prosthetic heart valves, the sensitivity analysis showed no changes in the USA regions associated with an increase or decrease in the odds of experiencing stroke/TIA combined or in-hospital all-cause mortality. However, odds ratios were reduced, resulting in a slight decrease in clinical risk for stroke/TIA and in-hospital all-cause mortality. For stroke/TIA combined, compared with the Northeast region, patients in the West were 60% more likely to experience a stroke or TIA (OR: 1.602, 95% CI: 1.096–2.342; p = 0.015). For in-hospital all-cause mortality, compared with the Northeast region, patients in the Midwest were 300% more likely to experience in-hospital death (OR: 4.009, 95% CI: 1.963–8.186; p < 0.001), and patients in the West were 428% more likely to experience in-hospital death (OR: 5.283, 95% CI: 2.594–10.763; p < 0.001). All USA regions were found to be statistically significant predictors for charges and LOS. After model adjustments were made, mean adjusted charges were slightly reduced, but rank order did not change. There were no changes in mean adjusted LOS.

Lastly, the final sensitivity analysis, completed using the TAVR cohort, revealed that use of the Sentinel CEP device (Boston Scientific) was associated with a reduced risk of stroke/TIA combined and in-hospital all-cause mortality. TAVR cases performed with CEP were 24.5% less likely to result in a stroke or TIA (OR: 0.755, 95% CI: 0.647–0.880; p < 0.001) and 26% less likely to result in in-hospital death (OR: 0.740, 95% CI: 0.596–0.918; p = .006). A generalized linear regression model was produced and confirmed that use of the Sentinel CEP device (Boston Scientific) was also a significant predictor of LOS.

## Discussion

This analysis provides a unique outlook on regional differences in outcomes for patients undergoing TAVR with the Sentinel CEP device. The current body of literature has focused on the questionable efficacy of CEP used in TAVR, comparing TAVR cases with and without CEP use. After controlling for covariates of interest, the West region of the USA had distinctively poorer outcomes for stroke, stroke/TIA combined, and in-hospital death. The Midwest region was also found to have higher odds of in-hospital death. The South region was identified as having relatively better outcomes, with patients in the south being less likely to experience a stroke. This finding was somewhat unusual. In general, health behaviors and outcomes, including stroke and heart disease mortality, are poorest in the South [28–31]. Further, the West is often associated with having better health behaviors and outcomes than other USA regions [28–31]. In an analysis of regional variation in TAVR outcomes, compared with the Northeast, patients in the Midwest and South were more likely to experience in-hospital death. The West was not significantly associated with in-hospital death [32]. Comparisons should not be made, though, as this analysis was performed using 2012–2013 data from the National Inpatient Sample. Not only did this study look solely at TAVR outcomes, but the time period is significant, as there have been impactful changes in valve generations between 2012 and 2019. There is a lack of USA regional studies resulting in an inability to compare or contextualize some of the results of this analysis.

In the TAVR cohort, after adjusting for covariates of interest, use of the Sentinel CEP device (Boston Scientific) was identified as a significant predictor of stroke/TIA combined, in-hospital death, and LOS. TAVR cases performed with the Sentinel CEP device (Boston Scientific) were less likely to result in stroke or TIA or in-hospital death. However, in the primary analysis of TAVR cases performed with the Sentinel CEP device, there is no apparent relationship between CEP utilization rates and outcomes. For example, the South and West regions had the lowest CEP utilization rates, and yet, patients in the South were less likely to experience stroke, while patients in the West were more likely. The Midwest had a relatively higher rate of CEP utilization; these patients were more likely to experience in-hospital death but had no significant association with stroke, TIA, or stoke/TIA combined.

After adjusting for covariates of interest, charges and LOS were highest in the West, which aligns with the poorer outcomes observed in this region. Additionally, regional differences in cost-of-living would be a significant contributor to the differences in charges. Charges should be interpreted with caution, as they are neither costs nor payments. However, the adjusted charges produced from this analysis can be a useful metric for making regional comparisons.

CEP utilization rates were consistent with previous studies [3,26,32]. Overall regional CEP use as a percent of the total CEP cohort (32.9% Northeast, 32.1% Midwest, 21.8% West, 13.2% South) was similar to the proportions reported from the 2018–2019 TVT registry, which included data from 599 TAVR sites (30.6% Northeast, 30.6% Midwest, 22% West, and 16.8% South) [3]. By the fourth quarter of 2019, 12.5% of cases were performed with the Sentinel CEP device. This rate is similar to the 13% CEP utilization rate also reported from the 2018–2019 TVT registry data [3]. The consistency between utilization rates reinforces the robust and regionally representative nature of the NIS data used to complete this analysis.

In this analysis, CEP utilization rates are likely the result of two influencing factors: reimbursement and clinical evidence. A year-over-year increase in CEP utilization was observed in each region. This finding coincides with the timing of reimbursement for the Sentinel CEP device (Boston Scientific), which occurred in October 2018, just over halfway through the study [11,15,16]. The overall low adoption and utilization of CEP, as observed by 2019 utilization rates, can be explained by the absence of clear and consistent efficacy data at the time; a trend that continues into the present.

The PROTECTED TAVR trial results were reported at the 34th Transcatheter Cardiovascular Therapeutics (TCT) conference, which occurred during the completion of this analysis. This global trial randomized 3,000 patients to TAVR with the Sentinel CEP device (Boston Scientific) or TAVR alone. Previous randomized trials were underpowered or failed to meet the primary outcome. While many hoped that the PROTECTED TAVR trial would provide a clear determination on device efficacy, the results have once again provided ambiguity. The trial did not meet its primary end point and revealed a non-significant lower rate of stroke in patients treated with the Sentinel CEP device (Boston Scientific). In the secondary analysis, however, a significant relative risk reduction in disabling stroke was observed in patients treated with the Sentinel CEP device (Boston Scientific) [33,34]. The results of this critical trial will have significant impact on future research in this space.

### Limitations

The limitations of this study include the potential for selection bias and residual confounding. Patients selected to receive CEP during TAVR may have an inherently higher risk of stroke. The STS risk factors for operative mortality and morbidity were considered for variable selection in the regression models and should have minimized the potential for selection bias [27]. Residual confounding is likely, due to the use of retrospective observational data. The NIS dataset did not include other predictors of stroke, including valve type (balloon expanding vs self-expanding), use of pre-dilation, and procedure time. In the SENTINEL trial, after adjusting for baseline lesion volume and valve type, a significant reduction in new lesion volume was identified in subjects who received the Edwards Sapien 3 balloon-expandable valve [35,36]. When the data from the SENTINEL trial was pooled with the data from the SENTINEL-H trial (Histopathology of Embolic Debris Captured During TAVR), the analysis revealed that total particle count and size were significantly higher in subjects treated with either the Evolut R valve (self-expanding) or Lotus valve (mechanically implantable) [37]. Other potential confounding variables may include implanter experience, hospital size, or teaching status. Model goodness-of-fit measures indicate overall poor-fitting models, which further supports the belief that unobserved characteristics are missing from this analysis. Further, this analysis has captured periprocedural strokes occurring prior to discharge. However, procedure-related stroke may occur up to the 30-day post procedure time point. Data collected from 2012 through 2019 for the TVT registry reveals that there is a slight increase in the rate of stroke from the in-hospital time point to the 30-day time point. As such, it is possible that there are procedure-related strokes that have occurred and are unaccounted for in this analysis [38]. Finally, additional limitations associated with the use of observational data include the potential for coding errors and the inability to determine the onset of events (i.e., comorbidity vs procedural complication).

## Conclusion

Within the USA, there are regional differences in the adoption and utilization of the Sentinel CEP device (Boston Scientific) during TAVR. Furthermore, there are regional differences in outcomes in TAVR procedures performed with the Sentinel CEP device (Boston Scientific). These outcomes, including stroke, stroke/TIA combined, in-hospital all-cause mortality, length of stay (LOS), and charges, do not appear related to CEP utilization rates. It has been speculated, after the presentation of the PROTECTED TAVR trial results, that CEP adoption will remain static [34]. Further research is needed to determine what variable(s) may be responsible for regional differences in TAVR outcomes, with or without CEP. This research is critical for preventing regional disparities and ensuring consistent quality of care in the USA. This analysis may be used for hypothesis formulation.

## Summary points

- The use of cerebral embolic protection (CEP), to mitigate the risk of stroke, during transcatheter aortic valve replacement (TAVR) remains low despite commercial availability since 2017 and reimbursement since 2018.
- While ongoing clinical studies are assessing the efficacy of the Sentinel and other investigational CEP devices, analysis on utilization trends remains sparse.
- Within the USA, there are regional differences in the utilization and outcomes of CEP use during TAVR.
- From 2017 through 2019, The Northeast had the greatest overall CEP utilization rate (11.3%), followed by the Midwest (11.1%), West (8.7%), then South (3.1%).
- The West region of the USA had poorer outcomes for stroke and stroke/TIA combined, compared with other USA regions.
- The West and Midwest regions of the USA were associated with a higher risk of in-hospital all-cause mortality, compared with other USA regions.
- Charges and length of stay were highest in the West.
- To prevent regional disparities and ensure consistent quality of care in the USA, further research is needed to determine what variable(s) may be responsible for regional differences in TAVR outcomes, with or without CEP.
