# Supplementary material for: Regional differences in the utilization and outcomes of cerebral embolic protection during transcatheter aortic valve replacement: an analysis of the National Inpatient Sample from 2017 through 2019
Source: J Comp Eff Res. 2023 Sep 19;12(10):e230010. doi: 10.57264/cer-2023-0010 (PMC10690440; doi:10.57264/cer-2023-0010)
Supplement: Supplementary file 1 [file cer-12-230010-s1.docx]

**Appendix Table 1** ICD-10 Codes

|  | Codes used |
| --- | --- |
| Inclusion | |
| Percutaneous TAVR | 02RF38Z |
| Transapical TAVR | 02RF38H |
| Sentinel cerebral protection device | X2A5312 |
| Exclusion | |
| Bicuspid Aortic Valve | Q230, Q231 |
| Outcomes | |
| Stroke | **Nontraumatic subarachnoid hemorrhage**: I60, I600, I6000, I6001, I6002, I601, I6010, I6011, I6012, I602, I603, I6030, I6031, I6032, I604, I605, I6050, I6051, I6052, I606, I607, I608, I609  **Nontraumatic intracerebral hemorrhage:** I61, I610, I611, I612, I613, I614, I615, I616, I618, I619  **Cerebral Infarction:** I63, I630, I6300, I6301, I63011, I63012, I63013, I63019, I6302, I6303, I63031, I63032, I63033, I63039, I6309, I631, I6310, I6311, I63111, I63112, I63113, I63119, I6312, I6313, I63131, I63132, I63133, I63139, I6319, I632, I6320, I6321, I63211, I63212, I63213, I63219, I6322, I6323, I63231, I63232, I63233, I63239, I6329, I633, I6330, I6331, I63311, I63312, I63313, I63319, I6332, I63321, I63322, I63323, I63329, I6333, I63331, I63332, I63333, I63339, I6334, I63341, I63342, I63343, I63349, I6339, I634, I6340, I6341, I63411, I63412, I63413, I63419, I6342, I63421, I63422, I63423, I63429, I6343, I63431, I63432, I63433, I63439, I6344, I63441, I63442, I63443, I63449, I6349, I635, I6350, I6351, I63511, I63512, I63513, I63519, I6352, I63521, I63522, I63523, I63529, I6353, I63531, I63532, I63533, I63539, I6354, I63541, I63542, I63543, I63549, I6359, I636, I638, I6381, I6389, I639 |
| TIA | G45, G450, G451, G452, G453, G454, G455, G456, G457, G458, G459 |
| Variables to include in regression analysis | |
| Anemia | D6489, D649, D50, D508, D509 |
| Atrial fibrillation or flutter | I48, I480, I481, I4811, I4819, I482, I4820, I4821, I483, I484, I489, I4891, I4892 |
| Chronic pulmonary disease | J449 |
| Coagulopathy | D689 |
| Carotid artery disease | I652, I6521, I6522, I6523, I6529 |
| Congestive heart failure | I50, I501, I502, I5020, I5021, I5022, I5023, I503, I5030, I5031, I5032, I5033, I504, I5040, I5041, I5042, I5043, I508, I5081, I50810, I50811, I50812, I50813, I50814, I5082, I5083, I5084, I5089, I509 |
| Peripheral vascular disease | I739 |
| Diabetes | **Type 1 DM:** E10, E101, E1010, E1011, E102, E1021, E1022, E1029, E103, E1031, E10311, E10319, E1032, E10321, E103211, E103212, E103213, E103219, E10329, E103291, E103292, E103293, E103299, E1033, E10331, E103311, E103312, E103313, E103319, E10339, E103391, E103392, E103393, E103399, E1034, E10341, E103411, E103412, E103413, E103419, E10349, E103491, E103492, E103493, E103499, E1035, E10351, E103511, E103512, E103513, E103519, E10352, E103521, E103522, E103523, E103529, E10353, E103531, E103532, E103533, E103539, E10354, E103541, E103542, E103543, E103549, E10355, E103551, E103552, E103553, E103559, E10359, E103591, E103592, E103593, E103599, E1036, E1037, E1037X1, E1037X2, E1037X3, E1037X9, E1039, E104, E1040, E1041, E1042, E1043, E1044, E1049, E105, E1051, E1052, E1059, E106, E1061, E10610, E10618, E1062, E10620, E10621, E10622, E10628, E1063, E10630, E10638, E1064, E10641, E10649, E1065, E1069, E108, E109  **Type 2 DM:** E11, E110, E1100, E1101, E111, E1110, E1111, E112, E1121, E1122, E1129, E113, E1131, E11311, E11319, E1132, E11321, E113211, E113212, E113213, E113219, E11329, E113291, E113292, E113293, E113299, E1133, E11331, E113311, E113312, E113313, E113319, E11339, E113391, E113392, E113393, E113399, E1134, E11341, E113411, E113412, E113413, E113419, E11349, E113491, E113492, E113493, E113499, E1135, E11351, E113511, E113512, E113513, E113519, E11352, E113521, E113522, E113523, E113529, E11353, E113531, E113532, E113533, E113539, E11354, E113541, E113542, E113543, E113549, E11355, E113551, E113552, E113553, E113559, E11359, E113591, E113592, E113593, E113599, E1136, E1137, E1137X1, E1137X2, E1137X3, E1137X9, E1139, E114, E1140, E1141, E1142, E1143, E1144, E1149, E115, E1151, E1152, E1159, E116, E1161, E11610, E11618, E1162, E11620, E11621, E11622, E11628, E1163, E11630, E11638, E1164, E11641, E11649, E1165, E1169, E118, E119 |
| Chronic kidney disease | N18, N181, N182, N183, N1830, N1831, N1832, N184, N185, N186, N189 |
| Obesity | E660, E6601, E6609, E668, E669  **BMI:** Z683, Z6830, Z6831, Z6832, Z6833, Z6834, Z6835, Z6836, Z6837, Z6838, Z6839, Z684, Z6841, Z6842, Z6843, Z6844, Z6845 |
| Hypertension | I10 |
| History of prior MI | I252 |
| History of PCI | Z955 |
| History of CABG | Z951 |
| History of stroke or TIA | Z8673 |
| History of smoking | Z87891 |
| Sensitivity Analysis | |
| History of prosthetic heart valve | Z952 |

*Note.* Obtained from *ICD10data* (n.d.)
